# Editorial Note: The Signal Sequence Coding Region Promotes Nuclear Export of mRNA

**DOI:** 10.1371/journal.pbio.3003822

**Published:** 2026-05-26

**Authors:** The PLOS Biology Editors

Concerns were raised that results and conclusions reported in this article [1] are unreliable based on findings reported in subsequent publications. The *PLOS Biology* Editors reviewed this matter and concluded that the experiments and data reported in [1] were in line with 2007 standards and remain scientifically valid, although the hypothesis and interpretation of the results reported in [1], including in the title, are no longer fully supported given knowledge that has since come to light [2–4], including evidence that some DNA reporter constructs had limitations [2]. The first and corresponding authors stated that the main conclusion, that GC-rich elements, such as the signal-sequence coding region, promote nuclear export of intronless mRNAs, remains valid and is supported by subsequent studies [5–6].

*PLOS Biology* stands by the article, but cautions readers not to rely on the conclusion expressed in the title, and to interpret the reported results in the context of current knowledge about mechanisms of mRNA export regulation.

Additionally, the corresponding author confirmed that Fig S6 is incorrect and is a duplicate of Fig S5, but stated that the original image underlying Fig S6, which the authors submitted to the journal at the time, is no longer available. PLOS considers that the results presented in Fig S6 are supported by other figures and do not significantly impact the overall conclusions of the article. The authors regret that the original data are no longer available.
